# Ad6-Based GM-CSF Expressing Vector Displays Oncolytic and Immunostimulatory Effects in an Immunocompetent Syrian Hamster Model of Cholangiocarcinoma

**DOI:** 10.3390/v17020162

**Published:** 2025-01-24

**Authors:** Daria S. Zabelina, Ivan D. Osipov, Denis E. Maslov, Anna V. Kovner, Valeriia A. Vasikhovskaia, Diana S. Demina, Stanislav E. Romanov, Ekaterina V. Shishkina, Julia Davydova, Sergey V. Netesov, Margarita V. Romanenko

**Affiliations:** 1 Faculty of Natural Sciences, Novosibirsk State University, Pirogov 2, Novosibirsk 630090, Russia; d.zabelina@g.nsu.ru (D.S.Z.); i.osipov6@g.nsu.ru (I.D.O.); d.maslov8@g.nsu.ru (D.E.M.); v.vasikhovskaia@g.nsu.ru (V.A.V.); d.demina@g.nsu.ru (D.S.D.); s.romanov@g.nsu.ru (S.E.R.); e.shishkina@g.nsu.ru (E.V.S.); netesov.s@nsu.ru (S.V.N.); 2 Institute of Cytology and Genetics, Siberian Branch of Russian Academy of Sciences (ICG SB RAS), 10 Akad. Lavrentiev Ave., Novosibirsk 630090, Russia; kovner@bionet.nsc.ru; 3 Department of Surgery, University of Minnesota, Minneapolis, MN 55455, USA; davyd003@umn.edu

**Keywords:** adenovirus type 6 (HAdV-C6, Ad6), oncolytic virus, cancer, cholangiocarcinoma, Syrian hamster, immunotherapy, liver cancer

## Abstract

Cholangiocarcinoma (CCA), the second most common liver cancer, remains highly resistant to chemotherapy and radiotherapy, leaving patients with unresectable tumors in urgent need of innovative therapeutic approaches. Adenovirus type 6 (Ad6), a species C human adenovirus, offers significant potential for cancer therapy due to its low seroprevalence compared to Adenovirus type 5 (Ad5) and its ability to evade Kupffer cells during systemic delivery. In this study, we developed a novel oncolytic adenovirus vector based on the Ad6 engineered to express human GM-CSF (Ad6-d24-GM) and evaluated its therapeutic efficacy in a novel immunocompetent, replication-permissive Syrian hamster model of CCA. Intratumoral administration of Ad6-d24-GM significantly suppressed tumor growth and prolonged survival without evidence of toxicity, as indicated by stable body weights and normal liver enzyme levels. Both Ad6-d24-GM and wild-type Ad6 induced robust infiltration of CD4+ and CD8+ T cells, as well as CD68+ macrophages within tumors, demonstrating activation of antitumor immunity. Notably, the Ad6-d24-GM group exhibited a statistically significant increase in CD68+ cells compared to wild-type Ad6, highlighting the immunomodulatory effect of GM-CSF transgene. These results demonstrate the oncolytic and immunostimulatory potential of Ad6-based vectors for CCA treatment and validate the Syrian hamster syngeneic CCA-OF model as a valuable platform for studying oncolytic adenovirus therapies.

## 1. Introduction

Cholangiocarcinoma (CCA), the second most common hepatic cancer, is frequently diagnosed at an advanced stage, leaving a large proportion of patients with unresectable tumors [1]. Additionally, CCA frequently exhibits resistance to both chemotherapy and radiotherapy, highlighting the need for the development of novel intervention strategies. Emerging immunotherapeutic approaches, including cancer vaccines, adoptive cell therapy, and immune checkpoint inhibitors (ICIs), are under active investigation and show promise for improving prognosis and overall survival in CCA patients [2]. Oncolytic virotherapy has emerged as a promising strategy, offering the potential to selectively target tumor cells while overcoming the resistance mechanisms that limit the efficacy of conventional therapies. Among these, oncolytic adenoviruses (OAds) are particularly notable due to their natural ability to efficiently infect epithelial cells, including cholangiocytes, inducing cell death through the adenoviral lytic cycle and promoting robust antitumor immune responses via immunogenic cell death. Furthermore, OAds are remarkably versatile, offering ease of genetic modification and a proven safety record demonstrated in clinical trials [3,4,5,6,7]. These qualities highlight the potential of OAds as a powerful therapeutic option for CCA, either as a monotherapy or in combination with existing modalities.

Several studies have demonstrated the ability of adenoviruses to successfully transduce and replicate in various human CCA cell lines [8,9,10]. The vectors used in these studies were based on human adenovirus type 5 (HAdV-C5, Ad5), which is highly seroprevalent in certain populations, potentially diminishing its therapeutic effectiveness [11]. To circumvent the neutralization issues associated with Ad5, alternative adenoviral platforms with lower seroprevalence are being explored. One promising candidate is human adenovirus type 6 (HAdV-C6, Ad6), which, while belonging to the same species C as Ad5 and sharing many of its biological properties, is significantly less seroprevalent. Importantly, Ad6 demonstrates a more efficient gene delivery in the liver compared to Ad5 when administered intravenously [12]. This enhanced liver tropism of Ad6 is attributed to its hexon protein, which evades macrophage and endothelial scavenger receptors, thus avoiding uptake by Kupffer cells, the liver macrophages [13,14,15]. Our group has previously reported the high oncolytic potential of Ad6-based vectors against glioblastoma and breast cancer [16,17], and other research groups have utilized the Ad6 backbone as an oncolytic agent against prostate cancer [18,19,20]. These unique features, combined with the demonstrated high oncolytic potential against solid tumors, position Ad6 as a more suitable vector for liver-targeted therapies in oncolytic virotherapy.

Although adenoviruses possess inherent antitumor activity, additional modifications are necessary to enhance their safety and boost their antitumor effects. The 24 bp mutation in the E1A gene ensures virus specificity for cells with a dysregulated Rb pathway, which is commonly observed in cancers of various origins, including CCA [21]. To further promote antitumor immunity, OAds are often engineered to express pro-inflammatory cytokines, such as Granulocyte-Macrophage Colony-Stimulating Factor (GM-CSF). GM-CSF plays a critical role in recruiting and activating antigen-presenting cells, thereby triggering both innate and adaptive immune responses against tumors [22]. Notably, GM-CSF has been successfully utilized in several oncolytic virus platforms, including Imlygic (talimogene laherparepvec, T-VEC), the first FDA-approved oncolytic virus based on herpes simplex virus type 1 (HSV-1), to enhance host immunity against cancer [3,23,24].

Most in vivo studies on OAds, including those for CCA, have been conducted using immunodeficient murine models with human xenografts, which do not allow for assessment of the viruses’ immunological activity. Syngeneic mouse models are not suitable for this purpose either, as murine tissues do not permit human adenovirus replication. This limitation prevents the assessment of both the immunostimulatory and oncolytic capabilities of human adenoviruses, as the ability to induce immunogenic cell death and the subsequent immunological effects cannot be evaluated in these models. However, animal models such as pigs, cotton rats, and Syrian hamsters are susceptible to human adenoviruses [25,26,27]. Syrian hamsters, in particular, are becoming the “gold standard” for OAds research due to their permissiveness for both coxsackie and adenovirus receptor (CAR)- and integrin-targeted vectors, as well as their ease of care and short reproductive cycles [28]. Additionally, several human cytokines, including GM-CSF, IL-2, and IL-21, are biologically active in hamsters, making them particularly suitable for evaluating immune responses and the effects of immunomodulatory transgenes in OAd studies [29]. Various syngeneic hamster tumor models, including pancreatic [29], kidney [28], glioblastoma [30], leiomyosarcoma [31], and gallbladder [32], have been used for OAds testing. However, the diversity and availability of hamster cancer cell lines remain limited compared to murine models. The recently established CCA-OF cell line, derived from hamster intrahepatic CCA associated with Opisthorchis felineus liver fluke infection, expresses potential human CCA biomarkers and exhibits morphological characteristics and phenotypes similar to those of human carcinomas [33].

In the current study, we developed an Ad6-based vector expressing human GM-CSF, Ad6-d24-GM, and examined its anti-tumor potential in a novel immunocompetent and viral replication-permissive Syrian hamster CCA-OF model. Our findings provide new insight into the therapeutic potential of Ad6-based vectors against cholangiocarcinoma and underscore the utility of the Syrian hamster model as a promising platform for evaluating oncolytic adenovirus therapies.

## 2. Materials and Methods

### 2.1. Viruses

An Ad6 genome fragment (1–2313 bp) was amplified and cloned into a ZrmI-linearized pBR322 plasmid using the blunt ligation method. The resulting plasmid was then modified using primers designed to loop out and delete 24 bases from 924 to 947 bp. The last plasmid vector was used to amplify the Ad6 DNA fragment (1–2313 bp), containing a 24 bp deletion for further recombination. The genomic DNA of a previously constructed Ad6-hT-GM [17] was digested using the CRISPR/Cas12a system to acquire the remaining Ad6 genome fragment (2291–35,579 bp) containing the human GM-CSF transgene in place of E3-6.7K/gp19K ORFs. The acquired DNA fragments, specifically designed to have 21 bp overlaps, were used in In-Fusion recombination (Takara Bio Inc., Shiga, Japan). The reaction mix was directly transfected into Ad293 cells using the Calcium phosphate Transfection Kit (Biospecifica, Novosibirsk, Russia) according to the manufacturer’s guidelines. A reaction mix containing only the Cpf1-digested adenovirus genomic DNA was transfected as a negative control. After 48 h, the cytopathic effect was observed in cells transfected with the recombination mix while absent in negative control cells. Medium from the infected cells was then transferred to A549 cells. Viral DNA was purified and screened for the expected genome structure by restriction enzyme digestion; the region across the 24 bp deletion was determined by Sanger sequencing. All PCR amplification steps were performed using CloneAmp polymerase (Takara Bio Inc., Shiga, Japan) following the manufacturers’ instructions. All primers and probes utilized for PCR and gRNA utilized for CRISPR/Cas12a digestion are listed in Table S1.

All viruses were propagated in A549 cells in Dulbecco’s modified Eagle medium, DMEM (Capricorn, Ebsdorfergrund, Germany), with 5% fetal bovine serum, FBS (Biowest, Nuaillé, France), and were harvested and purified via CsCl-gradient centrifugation. Viral prep was dialyzed using dialysis tubing 14 kDa MWCO (Viscase, Lombard, IL, USA) against sterile PBS and supplemented with sterile glycerol to a final concentration of 7%, aliquoted, and stored at −80 °C. TCID50 of a virus stock was determined by infection of Ad293 cells by serial 10-fold dilutions and calculated using the Spearman–Kärber method.

### 2.2. Cells Lines

The A549 (a human lung cancer) cell line was kindly supplied by The State Research Center of Virology and Biotechnology “Vector”. The U87 MG (a human glioblastoma astrocytoma) and HepG2 (a human hepatocellular carcinoma) cell lines were kindly gifted by the SPF-vivarium of the Institute of Cytology and Genetics SB RAS (Novosibirsk, Russia). The MDA-MB-231 (a human triple-negative breast adenocarcinoma) cell line was kindly provided by the Joint Center for genomic, proteomic, and metabolomics studies ICBFM SB RAS (Novosibirsk, Russia). The CCA-OF (a hamster cholangiocarcinoma cell line) was established and kindly provided by the Laboratory of Molecular Mechanisms of Pathological Processes of the Institute of Cytology and Genetics SB RAS (Novosibirsk, Russia). Normal human fibroblasts were kindly provided by the Laboratory of Developmental Genetics of the Institute of Cytology and Genetics SB RAS (Novosibirsk, Russia).

A549, HepG2, MDA-MB-231, U87 MG, normal human fibroblasts, and CCA-OF cells were cultured in DMEM (Capricorn Scientific, Ebsdorfergrund, Germany) supplemented with 5% FBS, 100 μg/mL streptomycin, 100 U/mL penicillin, and 250 ng/mL Amphotericin B at 37 °C and 5% CO_2_.

All cell lines were tested mycoplasma-free. All cells were used for experiments within 10 passages after thawing, and CCA-OFs were implanted for in vivo experiments within 4 passages after thawing.

### 2.3. Cell Viability Assay

The cytotoxic effect of Ad6-d24-GM and wild-type Ad6 (Ad6wt) was studied on a panel of human tumor cells: A549, HepG2, MDA-MB-231, U87 MG, and hamster cells CCA-OF. Additionally, the cytotoxic effect was determined for the non-cancerous fibroblast cell line. To determine viral dose-dependent activity, cells were seeded on a 96-well plate at a density of 10^4^ cells/well. The next day, cells were infected with viruses at the multiplicities of infection (MOIs) of 100, 10, 1, and 0.1 TCID50/cell for cancer cells and MOIs of 100, 10, and 1 for normal fibroblast cells. A mock medium was added for negative control. After an 8-day incubation, cell viability was estimated by the CyQUANT™ XTT Cell Viability Assay (Thermo Fisher Scientific, Waltham, MA, USA) according to the manufacturer’s guidelines. The optical density OD450/655 was measured on an iMark Microplate Absorbance Reader (BioRad, Hercules, California, DC, USA). The optical density in infected wells was calculated as % in relation to a negative control.

For human fibroblast cell line XTT assay was performed in three biological replicates to determine statistical differences between the effects of the viruses.

### 2.4. GM-CSF Expression

To assess transgene expression in vitro, A549 and CCA-OF cells were seeded on a 12-well plate at a density of 10^5^ cells/well. In 24 h, the cells were infected with Ad6-d24-GM with MOI = 10 TCID50/cell. A mock medium was added for negative control. The media samples were collected, and cells were pelleted by centrifugation at several time points (24 and 48 h post-infection); 100 μL of supernatant were probed for analysis. The GM-CSF content was measured using an ELISA kit for Colony Stimulating Factor 2 (Cloud-Clone Corp, Houston, TX, USA) according to the manufacturer’s guidelines.

### 2.5. Viral Replication Study

To determine viral replication dynamics, A549 (human) and CCA-OF (hamster) cells were seeded on a 12-well plate at a density of 10^5^ cells/well. In 24 h, the cells were infected either with Ad6wt or Ad6-d24-GM at MOI = 10 TCID50/cell. The cells and medium were collected by centrifuging at several time points (6, 24, 48 h post-infection). The lysis buffer from the Ribo-prep kit (Amplisens, Moscow, Russia) was added to the plate wells and then transferred to the pelleted cells. Total nucleic acid extraction was conducted according to the manufacturer’s recommendations.

To quantify the adenovirus DNA copy number, a hexon-specific qPCR was carried out using BioMaster HS-qPCR (Biolabmix, Novosibirsk, Russia) with the following conditions: initial denaturation at 95 °C for 5 min, 40 cycles of denaturation at 94 °C for 10 s, and an annealing/elongation step at 56 °C for 45 s. To quantify cell numbers, 18S rRNA gene-specific qPCR was carried out using HS-qPCR SYBR Blue (Biolabmix, Novosibirsk, Russia) with the following conditions: initial denaturation at 95 °C for 5 min, 40 cycles of denaturation at 94 °C for 10 s, and annealing/elongation step at 54 °C for 30 s. The approximating functions based on standard 10-fold dilutions of the Ad6, A549, and CCA-OF genomic DNA were plotted and used for the calculation of the target DNA template in the reaction. Viral DNA content in a cell was calculated by normalizing absolute hexon copies to absolute cell numbers in a sample. Quantitative PCR was carried out on the Gentier 96E real-time PCR system (Tianlong, Xi’an, China) and analyzed using the Gentier real-time PCR system Software v1.

### 2.6. Animals

Female Syrian hamsters (14 weeks old, ~100 g) were purchased from the SPF-vivarium of the Institute of Cytology and Genetics SB RAS (Novosibirsk, Russia). All experiments with animals were approved by the Animal Care and Use Committee of the Institute of Cytology and Genetics SB RAS (permission No. 172 7 May 2024) and were conducted according to federal and institutional regulations.

### 2.7. In Vivo Study

To establish cholangiocarcinoma allographs for the antitumor efficacy study, hamsters were injected subcutaneously with 10^6^ cells of CCA-OF cell line in 150 μL of PBS into the left flank. When tumors reached approximately 300 μL in size, the hamsters were randomized into groups based on tumor size (*n* = 6, 7), and treatment commenced. For intratumoral (i.t.) injections, each injection contained 50 μL of vehicle (PBS with 7% glycerol) or 3 × 10^9^ TCID50 of either Ad6wt or Ad6-d24-GM suspended in the vehicle and was injected in multiple locations throughout the tumor using a fan-like injection pattern. Two i.t. injections were performed in total, marked as the 0th and 2nd day of the experiment. Tumor growth was measured every two days with electronic calipers, recording the greatest length and width. Tumor volumes were calculated as 0.5 × length × width^2^. Animals were euthanized after tumors reached a volume of 2000 mm^3^ based on ethical considerations.

In a similar experiment, nine more hamsters were injected with CCA-OF and treated with PBS, Ad6wt, or Ad6-d24-GM (*n* = 3 for each group), as described above. All animals were sacrificed on the same date, 14 days post viral injection. The tumor, liver, spleen, kidneys, and lungs were weighed and exposed to 10% neutral formalin for future immunohistochemical analysis. Samples of the tumors and each organ (100 mg) were collected for the virus quantification and biodistribution study.

### 2.8. Viral Biodistribution

Tumor, liver, spleen, lung, and kidney samples were collected and snap-frozen in liquid nitrogen. A total of 50 mg of each sample was placed into a ceramic bead tube and homogenized in 100 μL PBS using the Bioprep-6 homogenizer (Allsheng, Hangzhou, China). Total nucleic acid extraction was conducted using a Ribo-prep kit (Amplisens, Moscow, Russia) according to the manufacturer’s instructions. The total amount of DNA in the samples was assessed on a QUBIT fluorometer (Thermo Fisher Scientific, Waltham, MA, USA). Quantitative PCR of adenovirus hexon was performed as described in Section 2.5.

### 2.9. Histopathological Analysis

The liver and tumor tissue samples exposed to 10% neutral formalin were dehydrated in a graded series of ethanol and xylene (STP-120, Thermo Scientific, Waltham, MA, USA). Dehydrated samples were enclosed in a paraffin medium HISTOMIX (BioVitrum, Saint Petersburg, Russia). For microscopic examination, sections of 3.5 μm thickness were prepared on a rotary microtome Microm HM 355S (Thermo Scientific, Waltham, MA, USA).

The resulting paraffin sections were stained via a standard protocol with hematoxylin and eosin (BioVitrum, Saint Petersburg, Russia). To determine the virus presence and immune response in tissue samples, immunohistochemical analysis was performed (immunohistochemical SpringBioScience kit HRP-125, Pleasanton, CA, USA) using specific primary antibodies to analyze to following:

1. Adenovirus (EMD Millipore Corp., #4034661, Temecula, CA, USA);
2. Epithelial CK19 (Abcam, #ab220193, Waltham, MA, USA);
3. Immune response (CD4 (Elabscience, #E-AB-F1105A, Houston, TX, USA), CD8a (Cloud-Clone Corp., #PAB099Ra01, Wuhan, Hubei, China), CD68 (Affinity, #DF7518, Buckingham, UK)).

The staining and the visualization were performed according to the manufacturer’s protocol and as described previously [34]. Quantitative analysis on hepatic histological sections was performed to assess the presence of hemorrhage, inflammation, and hepatocyte dystrophy (percentage from the field of view) using the morphometric method, as described above [35,36]. Using a closed test system for 100 points, the number of positive staining cells in the tumor tissue samples was determined by using the ImageJ software (version number 1.50i, https://imagej.net/ (accessed on 14 January 2025)).

### 2.10. Data Analysis

All statistical analyses were conducted using the R programming language (version 4.3.2) [37] with the lme4 [38] and lmerTest [39] packages for mixed-effect modeling in longitudinal analysis. A significance level of α = 0.05 was used for hypothesis testing, with appropriate adjustments applied to control for multiple tests.

To model longitudinal tumor growth data, we employed linear mixed effect modeling (LMM). We estimated the effect of the viral treatment on the tumor growth as the interaction term in the model volume ~ time + treatment + time:treatment + (1 + time|ID), where time, treatment, and their interaction are estimated as fixed effects and (1 + time|ID) is the term for random intercepts and slopes for time conditioned by individual animals. Statistical significance for the differences between the group-specific slopes (i.e., the interaction effect) was assessed by the Tukey’s range test, with Kenward–Roger approximation for degrees of freedom.

For time-to-death data, Kaplan–Meier curves were estimated and differences between groups were evaluated by the log-rank test. In addition, the Cox proportional hazard model was used to assess the relationship between the viral treatment group and the hazard of death. The Schoenfeld residuals were examined to validate the proportional hazards assumption.

Differences in cell viability, GM-CSF expression, and virus replication were tested by Welch’s *t*-test, followed by Bonferroni adjustment. The normality assumption was tested by the Shapiro–Wilk test.

For the Immunohistochemistry and histopathology data, the field-of-view percentages were averaged per animal for each marker analyzed, providing a single value per animal per marker. These averaged values were then compared across treatment groups by Welch’s ANOVA, followed by Tukey’s range test for post hoc comparisons. The interindividual variance homogeneity for each treatment group was validated by Levene’s test using the median as the location measure.

## 3. Results

### 3.1. Characterization of Ad6-d24-GM In Vitro

**Figure 1:**
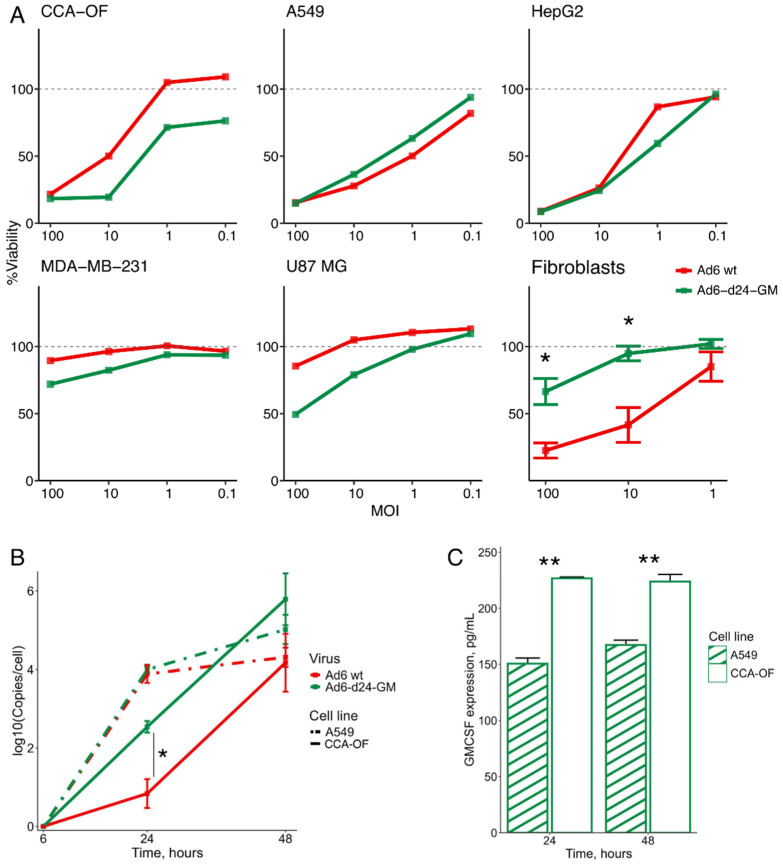
Characterization of Ad6-d24-GM in vitro. (**A**) The cytotoxic effects of Ad6-d24-GM were evaluated in a panel of human cancer cell lines (A549, HepG2, MDA-MB-231, U87 MG), hamster CCA-OF cells, and human non-cancerous fibroblasts. Cells were infected with either Ad6wt or Ad6-d24-GM at MOIs of 100, 10, 1, 0.1 TCID50/cell for cancer cells or MOIs of 100, 10, 1 TCID50/cell for normal fibroblasts. After an 8-day incubation cell viability was estimated by the XTT assay. Data are presented as means for technical replicates for human and hamster cancer cell lines. For normal fibroblasts, data were collected in three biological replicates, and the differences between cell viability levels were analyzed by Welch’s *t*-test. *p*-values were adjusted using the Bonferroni correction. (**B**) A549 (human) and CCA-OF (hamster) cells were infected with Ad6-d24-GM or Ad6wt (MOI = 10 TCID50/cell); cells and media were collected for DNA extraction at different time points (6, 24, 48 h post-infection) and adenovirus genome copy number was assessed by qPCR with primers targeting the Ad6 hexon gene. The 18S rRNA gene was used as a reference gene for normalization. The genome copy fold increase is plotted relative to the 6 hpi time point. Data are presented as mean ± standard error (*n* = 3). The differences between viral copy numbers for different viruses were analyzed at the point of 24 h in both cell lines by Welch’s *t*-test. *p*-values were adjusted using the Bonferroni correction. (**C**) Transgene expression in the supernatants of virus-infected A549 and CCA-OF cancer cell lines. Cells were infected with Ad6-d24-GM (MOI = 10 TCID50/cell); medium was collected, and GM-CSF concentration was measured by ELISA. Data are presented as mean ± standard error (*n* = 3). Expression levels were compared between two cell lines at each time point using Welch’s *t*-test. *p*-values were adjusted by the Bonferroni correction. Statistical significance is denoted as follows: * *p* < 0.05, ** *p* < 0.01 (adjusted *p*-values).

We engineered a novel oncolytic adenoviral vector, Ad6-d24-GM, based on Ad6, which contains a 24 bp mutation in the E1A gene and a replacement of the E3-6.7k/gp19k region with the human GM-CSF gene. First, we analyzed the cytotoxicity of the recombinant Ad6-d24-GM and compared its lytic effect to the wild-type Ad6 (Ad6wt). Cell viability was assessed in a panel of cancer cell lines: CCA-OF (cholangiocarcinoma, hamster), A549 (adenocarcinoma, human), HepG2 (hepatocarcinoma, human), MDA-MB-231 (breast cancer, human), and U87 MG (glioblastoma, human), as well as normal human fibroblasts (Figure 1A). Cells were infected with either Ad6-d24-GM or Ad6wt at various multiplicities of infection (MOIs), and cell viability was measured using the XTT assay 8 days post-infection. Ad6-d24-GM exhibited oncolytic activity in a dose-dependent manner in all the cell lines, similar to Ad6wt, suggesting that the 24 bp deletion and GM-CSF insertions did not reduce the virus’s lytic ability (Figure 1A). The A549 and HepG2 cells were the most sensitive to Ad6-d24-GM among the human cell lines. For the non-cancerous fibroblast cell line, an XTT assay was performed in three biological replicates, and statistical differences between the effects of Ad6-d24-GM and Ad6wt at different MOIs were analyzed. Ad6-d24-GM had a significantly lesser effect on cell viability compared to Ad6wt, which confirms that 24 bp modification promotes tumor-selectivity of the recombinant virus (Figure 1A, last panel).

To further evaluate the impact of the 24 bp deletion on viral replication, the human A549 and hamster CCA-OF cells were infected with either Ad6-d24-GM or Ad6wt (MOI = 10 TCID50/cell). Cells and media were collected at different time points (6-, 24-, and 48-h post-infection), and viral genome copy numbers were assessed by qPCR using primers specific to the Ad6 hexon gene (Figure 1B). The results show that Ad6-d24-GM exhibited faster growth rates in hamster CCA-OF cells compared to Ad6wt, as indicated by a nominal *p*-value of 0.03 at the 24 h post-infection time point. Otherwise, the replication rates of Ad6-d24-GM were comparable to Ad6, confirming that the recombinant virus retained its ability to replicate in CCA cells.

The secretion of GM-CSF following the infection of CCA-OF hamster cells with Ad6-d24-GM in vitro was confirmed by ELISA (Figure 1C). Notably, transgene expression was significantly higher in the CCA-OF cells compared to A549 cells. Therefore, Ad6-d24-GM was validated as an effective expression vector for the human GM-CSF transgene in Syrian hamster CCA-OF cells.

### 3.2. Ad6-d24-GM Suppressed Tumor Growth and Prolonged Survival in a Hamster Syngeneic Subcutaneous Tumor Model

**Figure 2:**
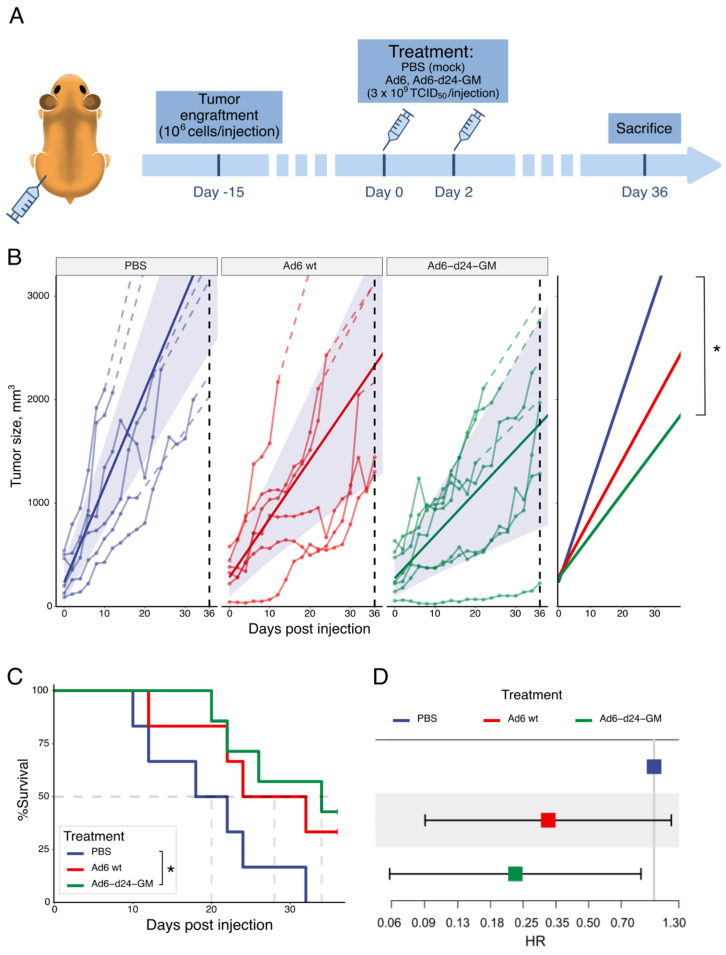
Oncolytic effect of viral treatment in a Syrian hamster syngeneic subcutaneous tumor model. (**A**) Experimental design. Established CCA-OF tumors (mean initial volume = 300 mm^3^) were injected with 3 × 10^9^ TCID50 of Ad6wt (*n* = 6), Ad6-d24-GM (*n* = 7) or PBS (*n* = 6) twice within a two-day interval. (**B**) Individual tumor growth trajectories over time across different treatment groups. Dashed lines show the trajectories after the sacrifice point imputed based on the best linear fit for each individual animal. Solid straight lines show tumor growth trends over time with shaded ribbons indicating 95% confidence intervals. The effect of the viral treatment was estimated as the interaction term coefficient for time and treatment group in the linear mixed effect. A pairwise comparison of the growth trends between treatment groups was conducted by a Tukey’s range test. The rightmost strip contains only trendlines for each of the treatment groups. (**C**) Survival curve of all experimental groups for the duration of the experiment. The dashed line indicates the median survival for each group. (**D**) Comparison of hazard rates in the Cox proportional hazard model for all of the treated groups. Grey solid line denotes 1. * *p* < 0.05 (adjusted *p*-value).

After showing a significant cytotoxic effect of Ad6-d24-GM in the CCA-OF cell line in vitro, along with its ability to replicate and express the human GM-CSF transgene, we assessed the tumor growth inhibition induced by Ad6-d24-GM in a novel cholangiocarcinoma hamster syngeneic subcutaneous model. Established CCA-OF tumors were injected with either Ad6-d24-GM, Ad6wt (3 × 10^9^ TCID50/injection), or a mock solution (PBS with 7% glycerol) twice within a two-day interval (Figure 2). We estimated the tumor growth rate in the viral treatment groups compared to the control using a linear mixed model (see Table S2 for the model parameters and statistics). The results showed that tumors treated with Ad6wt had their daily growth rate reduced by 36 mm^3^ compared to the control group, although this difference was not statistically significant. In contrast, Ad6-d24-GM treatment demonstrated a more pronounced effect, with a tumor growth rate of 41.48 mm^3^ per day compared to 92.83 mm^3^ per day in the PBS-treated control. This effect was statistically significant (nominal *p* = 0.0156, Tukey adjusted *p* = 0.0393) (Figure 2).

Furthermore, Ad6-d24-GM significantly increased the median survival to 34 days compared to 20 days in the PBS-treated group (*p* = 0.02, log-rank test). Mortality risk was further assessed using a Cox proportional hazards (PHs) analysis. Both viral treatments exhibited negative coefficients, suggesting a reduction in mortality risk compared to the control group. The hazard ratio (HR) for Ad6wt was 0.326, indicating a trend toward a 67.4% reduction in mortality risk, although this result was not statistically significant at alpha = 0.05 (*p* = 0.09). In contrast, the HR for Ad6-d24-GM was 0.23, suggesting a significant 77% reduction in the risk of death compared to the PBS-treated group (*p* = 0.03). These findings provide strong evidence supporting the therapeutic effect of Ad6-d24-GM.

### 3.3. Biodistribution and Safety

To investigate the spread of the virus and possible toxic side effects, we performed a separate endpoint experiment in hamster syngeneic subcutaneous tumors with the same setup as described previously. Animals were sacrificed at 14 dpi time point; blood, tumors, and organs were collected to assess immune response, biodistribution of the viruses, and possible toxicity to the organs. The 14-day time point was selected based on data from a preliminary study, in which we observed a clear therapeutic effect while ensuring that no animals in the control group showed signs of morbidity at day 14.

**Figure 3:**
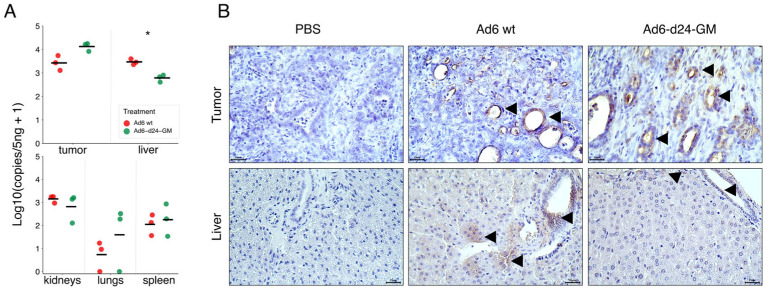
Adenovirus biodistribution. (**A**) Adenoviral genomic DNA biodistribution. Hexon gene copy number per 5 ng of genomic DNA from tumor, liver, kidney, lung, and spleen samples was evaluated by qPCR. Each dot represents the value of an individual animal; horizontal bars indicate the mean value for each group. The differences were analyzed by Welch’s *t*-test. *p*-values were adjusted using the Bonferroni correction. (**B**) Immunohistochemistry for Ad hexon in tumor and liver sections, magnification ×400 (scale bars = 25 μm). Groups of positive cells are indicated by arrows. * *p* < 0.05 (adjusted *p*-value).

Tumor, liver, lung, spleen, and kidney samples were assessed for viral genome copy number by qPCR (Figure 3A). Overall, the biodistribution patterns for both viruses were similar. As expected, the highest viral genome copy number was detected in the tumor samples for both Ad6-d24-GM and Ad6wt. Notably, the genome copy number of Ad6-d24-GM was elevated in tumors compared to Ad6wt while being significantly lower in liver samples (*p* = 0.02) These data suggest that Ad6-d24-GM replicates less in the liver compared to the wild-type virus. The same observation was supported by immunohistochemical staining for the adenoviral hexon protein (Figure 3B), although the results were not quantifiable. Interestingly, this IHC staining has also confirmed the possibility of Ad6 infecting intrahepatic bile ducts following intratumoral injection of the virus, as hexon-positive staining was observed in cells that could be morphologically identified as cholangiocytes.

**Figure 4:**
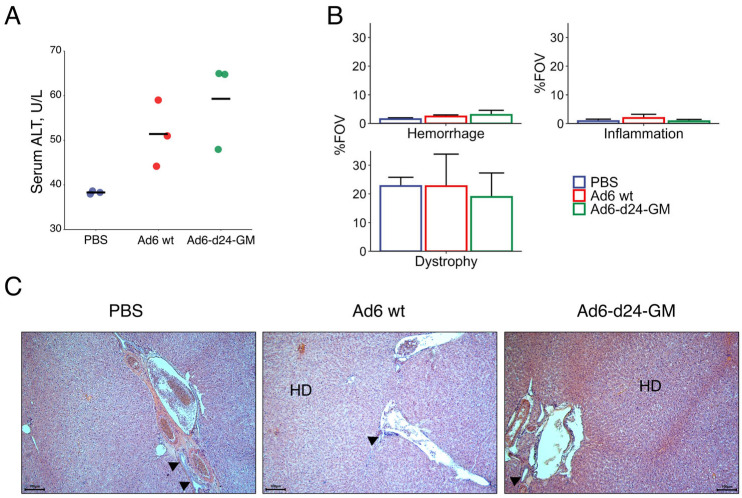
Intratumoral viral treatment caused no significant pathology in the liver. (**A**) Serum alanine transaminase levels. Each dot represents the value of an individual animal; horizontal bars indicate mean values for each group. (**B**) Quantitative changes in the liver, %FOV, indicating the percentage of the field of view occupied by the studied parameter. Data are presented as mean ± standard error (*n* = 3). The differences were tested by one-way ANOVA followed by Tukey’s range test for post hoc comparisons; *p*-values from the post hoc test were additionally adjusted for multiple testing by multiplying by the number of analyzed markers. No statistical significance was observed. (**C**) Representative images of the liver tissue; hematoxylin and eosin staining; magnification ×200 (scale bars = 100 μm). Portal triad ducts are indicated by arrows; HD—hepatocyte dystrophy.

No significant differences were observed in the body mass dynamics or the organ weight-to-body mass ratio between the groups. Serum alanine transaminase (ALT) levels were measured to assess possible toxicity to the liver (Figure 4A). Even though ALT was elevated for both of the groups receiving viral injections, the obtained values stayed in the normal range of 20–128 U/L [40]. Histopathological assessment of liver tissue showed no significant differences between the groups in any of the examined parameters: hepatocyte dystrophy, hemorrhages, and inflammatory infiltrate (Figure 4B,C). It is safe to assume that intratumoral injections of Ad6-d24-GM can significantly suppress tumor growth without serious side toxicity.

### 3.4. Treatment with Ad6-d24-GM Promotes T-Lymphocyte and CD68+ Macrophage Infiltration

**Figure 5:**
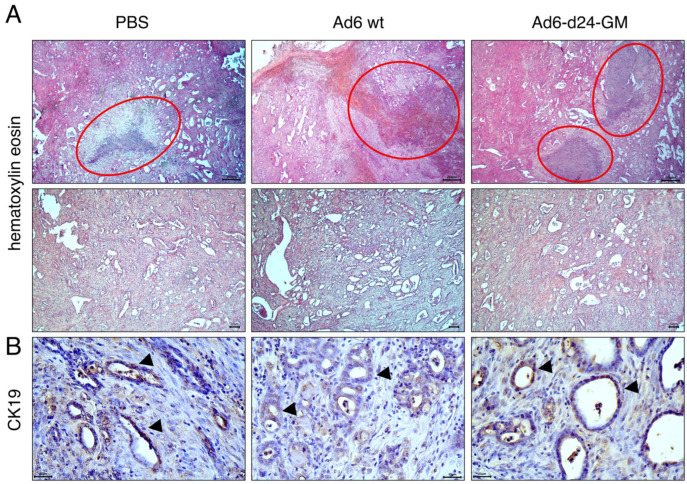
Histopathological assessment of tumor tissue obtained from the end-point experiment. (**A**) Representative images of tumor tissue; hematoxylin and eosin staining. Magnification ×50 (top, scale bars = 250 μm), ×100 (bottom, scale bars = 50 μm). Areas of necrosis are highlighted with red circles. (**B**) Immunohistochemistry of tumor tissue for cytokeratin 19. Arrows indicate positive staining. Magnification ×400 (scale bars = 25 μm).

Tumor tissue obtained from the end-point experiment was assessed by immunohistochemistry to determine immune response to the treatment. In all study groups, the tumor tissue was highly differentiated, with clearly visualized epithelial cells. The CCA origin was further confirmed by IHC staining for the duct marker cytokeratin 19 (CK19). Notably, the areas of destruction in the tumor tissues of animals from the groups receiving viral treatment were visually increased (Figure 5).

**Figure 6:**
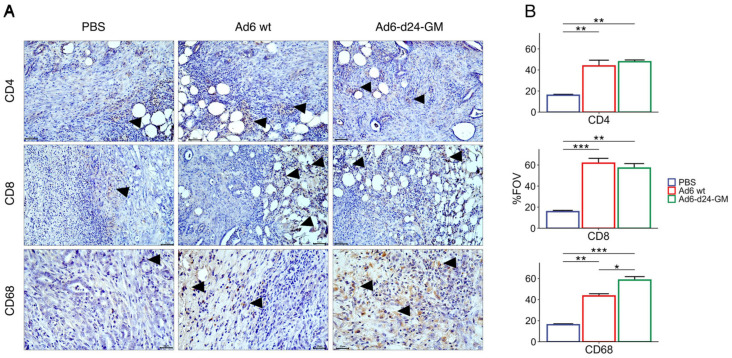
Immunohistochemical assessment of the immune response in tumor tissue. (**A**) IHC staining of tumor tissue with CD4, CD8, and CD68 antibodies. Positive cells are highlighted by arrows. Magnification ×400 (scale bars = 25 μm). (**B**) Quantitative changes in immune response; %FOV indicating the percentage of the field of view occupied by the studied parameter. Data are presented as mean ± standard error (*n* = 3). The differences were analyzed by one-way ANOVA followed by Tukey’s range test for post hoc comparisons. *p*-values from the post hoc test were additionally adjusted for multiple testing by multiplying by the number of analyzed markers. Statistical significance is denoted as follows: * *p* < 0.05, ** *p* < 0.01, *** *p* < 0.001 (adjusted *p*-values).

The immunohistochemical assessment of the immune response in tumor tissue showed an increased number of CD4+ and CD8+ cells in both Ad6-d24-GM and Ad6wt groups compared to the PBS-treated group (*p* < 0.01 for CD4+ and CD8+ in both treatments compared to PBS), reflecting higher infiltration of T-lymphocytes in response to viral treatment (Figure 6). The same was observed for CD68+ staining, the tumor-associated macrophages (TAMs) marker. Interestingly, Ad6-d24-GM led to significantly higher CD68 expression in tumors compared to the Ad6wt (*p* < 0.05), which may be attributed to the GM-CSF transgene effect.

## 4. Discussion

Cholangiocarcinoma (CCA) is an aggressive, poorly diagnosed cancer that often exhibits resistance to both chemotherapy and radiotherapy [41]. While some immunotherapeutic strategies, such as immune checkpoint inhibitors and chimeric antigen receptor T cells (CAR T cells), have shown promising results against CCA [42,43,44], the complex immune microenvironment limits their efficacy [45].

Oncolytic viruses represent another promising class of immunotherapy that can potentially overcome the limitations of traditional chemotherapies and immune checkpoint blockade. Adenoviruses are considered among the most promising vectors for oncolytic therapy due to their well-established replication machinery, genetic stability, high gene transfer efficiency, ease of production, and ability to selectively infect and kill cancer cells while stimulating antitumor immunity. The safety and efficacy of oncolytic adenoviruses (OAds) against various malignancies have been confirmed in numerous clinical trials [3,4,5,6,7]. Despite these advantages, research into OAds for CCA has been limited, with early studies demonstrating adenovirus-mediated apoptosis and cytotoxicity in CCA cells [8,46,47]. Recent advancements, such as the enhancement of immune responses in photodynamic immunotherapy, have further augmented OAds’ therapeutic potential [10].

One key challenge in evaluating OAds for CCA has been the reliance on immunodeficient models, which fail to capture the immunotherapeutic effects of the viral treatment. In this study, we addressed this limitation by using a unique immunocompetent, replication-permissive Syrian hamster CCA model, which allowed for a comprehensive evaluation of both the oncolytic and immunostimulatory effects of our novel Ad6-based vector.

Previous studies from our group and others have demonstrated that Ad6 is a potent oncolytic agent with distinct advantages over the more commonly studied Ad5 vector [16,17,48,49]. First, Ad6 is significantly less seroprevalent than Ad5, making it a more viable option for therapeutic use. In the US and Europe, Ad6 seroprevalence ranges from 8.5 to 45.7%, compared to 38.0–69.1% for Ad5 [11]. Importantly, anti-Ad5 neutralizing antibodies do not exhibit cross-reactivity with Ad6, further highlighting its potential to evade pre-existing immunity [50]. Second, unlike Ad5, Ad6 is able to evade uptake by Kupffer cells in the liver, resulting in a significantly higher transduction efficiency in hepatocytes and potentially cholangiocytes, as these cells are epithelial and naturally susceptible to adenovirus infection [8,13,46,47]. Structural differences in the hexon protein, particularly in the hypervariable regions, contribute to Ad6’s reduced recognition by scavenger receptors, which may explain its ability to escape capture by tissue-resident macrophages. These features make Ad6 a particularly attractive candidate for liver-directed therapies, including intravesical treatment of CCA.

Considering these advantages, we generated Ad6-d24-GM, a novel Ad6-based adenovirus expressing GM-CSF and featuring a 24-bp deletion in the CR2 domain of the E1A gene. The 24 bp modification enables tumor-specific replication by relying on the inactivation of the Rb protein pathway [51], whose disruption in CCA has been demonstrated in both human patients [52] and hamster models [53]. The use of the 24 bp deletion in OAds has been intensively studied, with numerous reports, including Phase 1 and 2 clinical trials, confirming its safety and tumor selectivity [54,55].

We confirmed the tumor-selective replication of Ad6-d24-GM by demonstrating its significantly reduced cytotoxicity in normal human fibroblasts compared to wild-type Ad6 (Ad6wt), while maintaining equal or even superior cytotoxicity in various human cancer cell lines, including a liver cancer cell line.

To assess the antitumor activity of Ad6-based oncolytics in immunocompetent hamsters, we used a recently developed hamster intrahepatic cholangiocarcinoma cell line, CCA-OF. It is a tumorigenic cell line derived from tumors in hamsters caused by Opisthorchis felineus infection. It was previously demonstrated that allografts established with CCA-OF express human CCA biomarkers, making it an adequate model for human CCA [33].

The hamster CCA-OF cell line was found to be sensitive to Ad6-based vectors, exhibiting cytotoxicity at a level comparable to or even surpassing that of certain human cancer cell lines. This confirms the potential of employing CCA-OF as a model for studying Ad6 oncolytics.

Another modification that was performed was the insertion of human GM-CSF at the E3-6.7k/gp19k region. As Syrian hamsters are susceptible to human GM-CSF [56], the effect of this cytokine can be successfully assessed in the hamster model. When evaluating early viral replication dynamics, Ad6-d24-GM exhibited faster growth rates in CCA-OF cells compared to Ad6wt and successfully expressed the GM-CSF transgene. These results validate Ad6-d24-GM as an effective expression vector.

In a CCA-OF syngeneic tumor model, both Ad6-d24-GM and Ad6wt resulted in tumor growth inhibition after only two doses. Notably, the tumor suppression by Ad6-d24-GM was more prominent and increased survival. To better understand the mechanisms behind tumor growth inhibition, we analyzed the immune response in tumor tissues following intratumoral injections. We observed a significant increase in CD68+ staining, a marker for macrophages, in the Ad6-d24-GM group compared to Ad6wt. GM-CSF is known to influence the tumor microenvironment by recruiting and activating monocyte-derived cells that present tumor antigens [57]. However, GM-CSF can sometimes exhibit pro-tumorigenic properties by promoting macrophage polarization to the immunosuppressive M2 phenotype [57]. OAds are known to reshape the immunological landscape and can switch macrophages back from an anti- to pro-inflammatory phenotype [58,59]. Based on significant tumor control following Ad6-d24-GM injection, we can speculate that adenoviral infection can overcome immunosuppressive macrophage polarization and even turn them in favor of the therapy. Further immunophenotyping of infiltrating cells could provide deeper insight into the mechanisms of immunomodulation. We also observed elevated CD4+ and CD8+ T cell infiltration in the tumor tissue of both viral-treated groups, which suggests that the Ad6 backbone, even without cytokine activity, may lead to the induction of an immune response in the tumor. These data indicate the possibility of Ad6 enhancing the efficacy of other immunotherapy approaches. The novel CCA-OF model could potentially be employed to investigate the combined OAd therapy with checkpoint inhibitors or adoptive cell therapies.

Intratumoral injection of viruses can lead to their distribution to distant organs via the bloodstream due to increased tumor vascularization. Indeed, adenovirus DNA was detected in all studied organs. Despite similar biodistribution patterns, Ad6-d24-GM was found at a higher concentration in the tumor and was significantly less present in the liver compared to Ad6, suggesting reduced hepatotropism due to genome modification. Liver tropism and toxicity are not major concerns for human therapy, as it has not been reported in clinical trials for systemically delivered OAds [60,61,62]. However, in rodents, the liver is an appropriate organ for evaluating OAd toxicity due to its increased sensitivity to the virus. We observed a slight elevation in serum ALT in both viral groups, but it was within the normal range [40]. Histopathological examination of the liver tissue showed no signs of pathology after either treatment. Therefore, intratumoral injections of Ad6-d24-GM can significantly suppress tumor growth and induce antitumor immunity without causing serious toxicity. Further research is required to establish the optimal dosing regimen to maximize the therapeutic effect.

Intrahepatic carcinoma is anatomically challenging to access, and systemic drug delivery is often more convenient for treating internal organ cancers [63]. There is some evidence suggesting that adenoviruses, particularly Ad2, the same species C as Ad6 and Ad5, can infect bile duct epithelium [64] upon gastrointestinal disease. We also detected cholangiocytes stained for Ad6 hexon in histological samples, suggesting the potential for systemic drug delivery or hepatic vessel administration. Further studies are needed to assess the toxicity and antitumor effects of systemic Ad6 injection.

## 5. Conclusions

In summary, we generated a recombinant oncolytic adenovirus (Ad6-d24-GM) and evaluated its therapeutic potential in a novel permissive cholangiocarcinoma hamster model. We demonstrated that intratumoral administration of Ad6-d24-GM inhibited tumor growth and induced T cells and macrophage infiltration without causing significant liver toxicity, suggesting its potential as a therapeutic option for CCA. We also highlighted the novel CCA-OF Syrian hamster model as a promising preclinical platform to assess OAds’ oncolytic and immunostimulatory effects.

## Data Availability

Processed data are contained within the article. Raw data are available upon request.

## Supplementary Materials

The following supporting information can be downloaded at https://www.mdpi.com/article/10.3390/v17020162/s1, Table S1: Primers, gRNA, and probes used in this study; Table S2: Regression statistics and estimates.
