# Supplementary material for: Ad6-Based GM-CSF Expressing Vector Displays Oncolytic and Immunostimulatory Effects in an Immunocompetent Syrian Hamster Model of Cholangiocarcinoma
Source: Viruses. 2025 Jan 24;17(2):162. doi: 10.3390/v17020162 (PMC11861176; doi:10.3390/v17020162)
Supplement: Supplementary file 1 [file viruses-17-00162-s001.zip › viruses-3399101-supplementary.pdf]

Table S1. Primers, gRNA and probes used in the study

Name	Sequence 5′ → 3′	Designation
Cpf_lin_for	CATCATCAATAATATACCTTATTTTGGATTGAAGCCAATATG	Primers used to generate fragment 1-2313 of Ad6 genome both to insert a fragment into pBR322 plasmid and to recombine with the remaining genomic DNA
Cpf_lin_rev	GCCCCTGCCCATCCTCGTTAATGGTTAAAATG	
Delta24-for	ATCGATCACCTCCGGCACAAGGTTT	Primers used to generate d24-mutated variant of E1A
Delta24-rev	CCACCCAGTGACGACGAGGATGAA	
VV3	AAAUUUCAUCUGUUUGUAGAUGCCCCUGCCCAUCCTCGUUAAU	gRNA used to excise 1-2313 fragment of adenovirus genome DNA
18s_1001f_ds	CTCAACACGGGAAACCTCAC	Primers used to quantify cell 18s rRNA copy number
18s_1110r_ds	CGCTCCACCAACTAAGAACG	
Ad6hex_f	gCCATTACTTTTgACTCTTCTgT	Primers and probe were used to quantify adenovirus hexon copy number
Ad6hex_r	CtgCTgATAATCTTTgTATTTAgTATC	
Ad6hex_pr	[FAM]AgAAACTTCCAgCCCATgAgCCg[BHQ1]	

Table S2: Regression statistics and estimates

Fixed effects					
	Estimate	Std.Error	DF	t	p
intercept	232.00	98.19	16.96	2.363	0.0304
time	92.83	14.20	17.26	6.537	$4,70*10^{-6}$
Ad6wt	51.49	137.59	16.36	0.374	0.7131
Ad6-d24-GM	41.40	132.28	16.24	0.313	0.7583
time × Ad6wt	-36.00	19.81	16.48	-1.817	0.0875
time × Ad6-d24-GM	-51.34	19.02	16.29	-2.699	0.0156
Marginal R^2 / Conditional R^2	0.386/0.967				
**Random effects**					
σ^2			$2.93*10^4$		
$\tau_{00\ ID}$			$4.81*10^4$		
$\tau_{11\ ID,\ time}$			$1.09*10^3$		
$\rho_{01\ ID}$			$7.00*10^{-1}$		
ICC			$9.50*10^{-1}$		
